# ﻿*Oreonectes qinae* (Teleostei, Cypriniformes, Nemacheilidae), a new loach species from Guangxi, China

**DOI:** 10.3897/zookeys.1255.158447

**Published:** 2025-10-15

**Authors:** Zhuo-Ni Chen, Cai-Huan Mo, Li-Na Du, Li-Na Zhang

**Affiliations:** 1 Key Laboratory of Ecology of Rare and Endangered Species and Environmental Protection (Guangxi Normal University), Ministry of Education, Guilin, Guangxi 541004, China; 2 Guangxi Key Laboratory of Rare and Endangered Animal Ecology, College of Life Sciences, Guangxi Normal University, Guilin, Guangxi 541004, China; 3 Forest Resources and Ecological Environment Monitoring Center of Guangxi Zhuang Autonomous Region, Nanning, Guangxi 530022, China

**Keywords:** Cavefish, cryptic species, *Cytb*, karst, Liujiang River, mitochondrial gene, *
Oreonectes
*, subterranean, taxonomy

## Abstract

A new species of loach, *Oreonectes qinae***sp. nov.**, was collected in December 2024 from Guangxi Gutingshan Forest Park, Liuzhou City, Guangxi Zhuang Autonomous Region, China. The new species can be distinguished from all other congeners by a suite of morphological characters, including a dorsal-fin origin posterior to the pelvic-fin origin, a degenerated posterior chamber of the swim bladder, and an incomplete lateral line, with 11–13 lateral-line pores. Phylogenetic analysis based on mitochondrial cytochrome *b* (*Cytb*) gene sequences confirmed its separation, with uncorrected *p*-distances ranging from 1.4% to 8.7% compared to closely related species. The discovery of *Oreonectes qinae***sp. nov.** reflects the hidden diversity within *Oreonectes* in the complex karst landscapes of Guangxi and emphasizes the need for further investigation and conservation of these cryptic freshwater species.

## ﻿Introduction

The genus *Oreonectes* Günther, 1868 includes a group of loaches predominantly distributed across southwestern China, including Guangxi Zhuang Autonomous Region, Guangdong, and Hong Kong, as well as Quang Ninh Province in northern Vietnam (Zheng 1981; Zhu and Cao 1987; Zhu 1989; Lan et al. 1995; Kottelat 2001; Zhong et al. 2024). Established with *Oreonectes platycephalus* Günther, 1868 as the type species, the genus is morphologically characterized by a flattened head; anterior and posterior nostrils separated by a distance shorter than the diameter of the posterior nostril; anterior nostril elongated into a barbel-like projection exceeding the nostril tube depth; longitudinal black stripe extending from the upper margin of the gill opening or dorsal fin to the middle of the caudal-fin base; six to seven branched dorsal-fin rays; rounded caudal fin; caudal peduncle elongated and narrow; and having an epural in the caudal skeleton (Günther 1868; Sawada 1982; Zhu and Cao 1987; Du et al. 2008; Prokofiev 2010).

Rapid tectonic uplift associated with the eastern Tibetan Plateau (Su et al. 2019a; Spicer et al. 2021) has dramatically altered the geomorphology of southwestern China, fragmenting subterranean habitats and generating isolated aquatic systems nested within intricate karst networks (Chen and Yu 1985). These environmental shifts promoted allopatric speciation, while intermittent surface water connectivity permitted episodic gene flow and secondary contact among populations (Luo et al. 2021; Wen et al. 2022). Within this context, *Oreonectes* species have largely specialized for life in underground rivers and surface streams within the karst region of southwestern China (Huang et al. 2009; Liu et al. 2019). Several taxa, including *O. polystigmus* Du, Chen & Yang, 2008, *O. guananensis* Yang, Wei, Lan & Yang, 2011, *O. luochengensis* Yang, Wu, Wei & Yang, 2011, and *O. andongensis* Luo, Du, Yang & Luo, 2024, have been collected from cave environments; while others, such as *O. platycephalus*, *O. guilinensis* Huang, Yang, Wu & Zhou, 2020, *O. damingshanensis* Yu, Luo, Lan, Xiao & Zhou, 2023, *O. zhangi* Zhong, Yang, Mo & Chen, 2024, and *O. yuedongensis* Luo, Lan, Xiao & Zhou, 2024, mainly inhabit surface freshwater rivers or streams. Although *O. platycephalus* was once considered widespread in Guangxi and Guangdong, integrative morphological and molecular analyses have revealed the presence of several cryptic species within this assemblage (Lan et al. 2024). Newly delineated species, such as *O. damingshanensis* (Xijiang River, Guangxi), *O. zhangi* (Nanliujiang, Guangxi), and *O. yuedongensis* (Pearl River, Guangdong), highlight significant hidden diversity within the genus (Zhu 1989; Du et al. 2008; Huang et al. 2020; Yu et al. 2023; Lan et al. 2024; Luo et al. 2024; Zhong et al. 2024). This high cryptic species diversity is likely due to the fragmented karst landscapes, combined with the limited dispersal capacities of benthic freshwater fish, promoting geographic isolation and genetic divergence among populations (Luo et al. 2021; Wen et al. 2022; Lan et al. 2024). At present, nine species of *Oreonectes* are recognized, including *O. platycephalus*, *O. polystigmus*, *O. guananensis*, *O. luochengensis*, *O. guilinensis*, *O. damingshanensis*, *O. andongensis*, *O. zhangi*, and *O. yuedongensis*.

In December 2024, 14 specimens were collected from small tributary streams in the Gutingshan Forest Park, Youfeng District, Liuzhou City, Guangxi Zhuang Autonomous Region, China. Based on a combination of morphological and molecular evidence, these specimens were determined to represent a previously unrecognized species of *Oreonectes*. This study provides a comprehensive morphological description of the new species and comparative analyses with its congeners, contributing to a deeper understanding of the biodiversity and evolutionary history of *Oreonectes* in the karst ecosystems of southern China.

## ﻿Material and methods

All experimental procedures complied with the Implementation Rules of the Fisheries Law of the People’s Republic of China and the Laboratory Animal Guidelines for the Ethical Review of Animal Welfare (GB/T 35892–2018). Fourteen type specimens were deposited in the Kunming Natural History Museum of Zoology, Kunming Institute of Zoology (KIZ), Chinese Academy of Sciences (CAS). Among them, six specimens were preserved in 10% formalin, while eight specimens were preserved in 99% ethanol. For DNA extraction, the right pelvic fin of each individual was removed, preserved in 99% ethanol, and stored in a −20 °C freezer. Morphological data were measured point-to-point with a vernier caliper (accuracy of 0.1 mm), following the measurement protocols outlined by Kottelat (1990). Measurements were recorded from the left side of each specimen and processed using Microsoft Excel. Standard abbreviations include SL (standard length), TL (total length), and HL (lateral head length).

Genomic DNA was extracted from ethanol-preserved fin tissues using a DNA extraction kit (Sangon Biotech (Shanghai) Co., Ltd., China). The mitochondrial cytochrome *b* (*Cytb*) gene was amplified using the primer pair F14724 (5’-GACTTGAAAAACCACCGTTG-3’) and R15915 (5’-CTCCGATCTCCGGATTACAAGAC-3’) following the protocols of Xiao et al. (2001). Polymerase chain reactions (PCR) were carried out in a 25 µL reaction volume with the following cycling conditions: an initial denaturing step at 98 °C for 3 min, 37 cycles of denaturing at 98 °C for 10 s, annealing at 55 °C for 15 s and extending at 72 °C for 15 s, and a final extension at 72 °C for 5 min. Sequencing of amplified products was performed by Sangon Biotech (Shanghai) Co., Ltd (China). Uncorrected *p*-distances were calculated using the Kimura2-parameter (K2-P) model and 1000 bootstrap replicates in MEGA v. 7.0 (Kumar et al. 2016), based on the *Cytb* gene. Two-way sequence reads were assembled and refined using SeqMan in DNAStar, while other sequences were downloaded from GenBank, with sequence alignment performed in MEGA v. 11.0 (Tamura et al. 2021). The newly generated sequences were deposited in GenBank (Accession Nos. PV266529–PV266532).

The phylogenetic position of *Oreonectes qinae* sp. nov. was inferred using Bayesian inference (BI) methods, implemented via the CIPRES Science Gateway (Miller et al. 2010). BI analysis was conducted using MrBayes in XSEDE v. 3.2.7a (Ronquist et al. 2012), employing two runs and four Markov chains starting from a random tree. Chains were run for seven million generations and sampled every 100 generations, with the first 25% of sampled trees discarded as burn-in. The optimal nucleotide substitution model for *Cytb* was determined under the AICc criterion, using PartitionFinder v. 2.1.1, identified as GTR+I+G. The remaining trees were used to construct a consensus tree and estimate Bayesian posterior probabilities (BPPs). The constructed phylogenetic trees were viewed and edited using FigTree v. 1.4.4 (Rambaut 2009).

## ﻿Results

### ﻿Taxonomy

#### ﻿ Oreonectes qinae

Taxon classificationAnimaliaCypriniformesNemacheilidae

Chen, Mo, Zhang & Du sp. nov.

51D6BE3A-2C00-5A45-BA21-1CDF035877D5

https://zoobank.org/9FF0EFA1-192D-45F7-A439-7D3E06679A07

**Figure 1.**
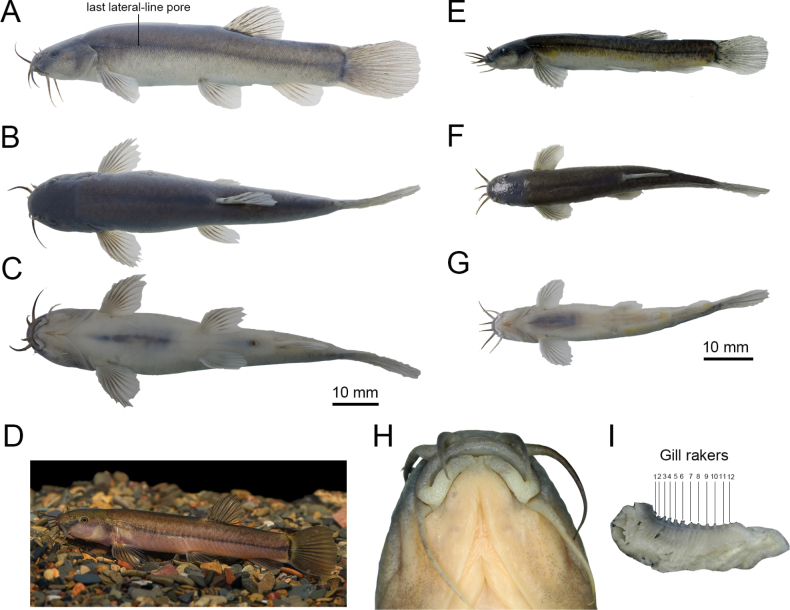
*Oreonectes qinae* sp. nov. A–C. Lateral, dorsal, and ventral views of holotype KIZ20240559 (♀); D. Live photo of female, photo by Zhou Jia-Jun; E–G. Lateral, dorsal, and ventral views of paratype KIZ20240564 (♂); H. Ventral view of head of holotype KIZ20240559 (♀) I. Photo of the gill rakers.

**Figure 2.**
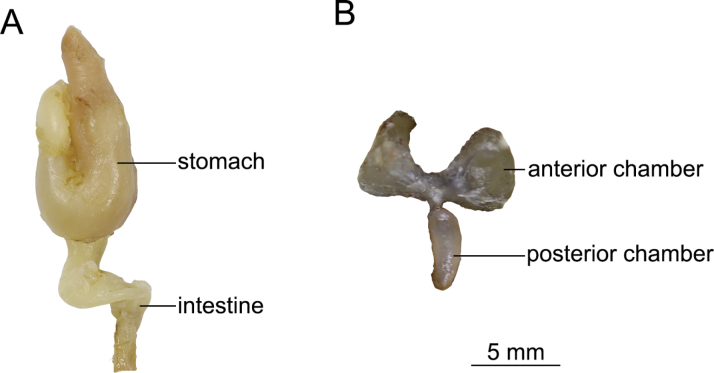
Morphological characteristics of paratype KIZ20240563 (♂) of *Oreonectes qinae* sp. nov., stomach and intestine (A); Anterior and posterior chambers (B).

**Figure 3.**
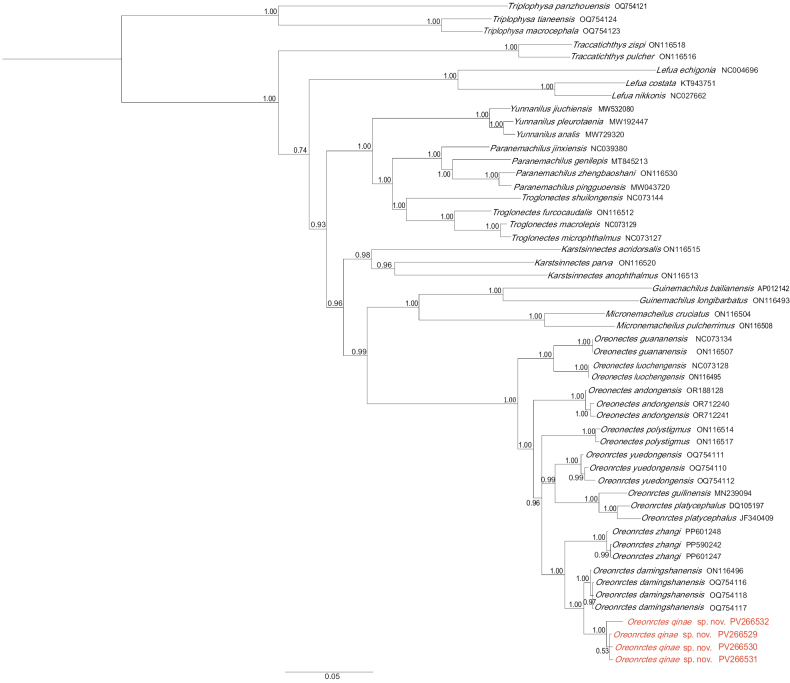
Bayesian phylogram of *Oreonectes* based on cytochrome *b* (*Cytb*) sequences. Numbers on branches represent BPPs from BI.

**Figure 4.**
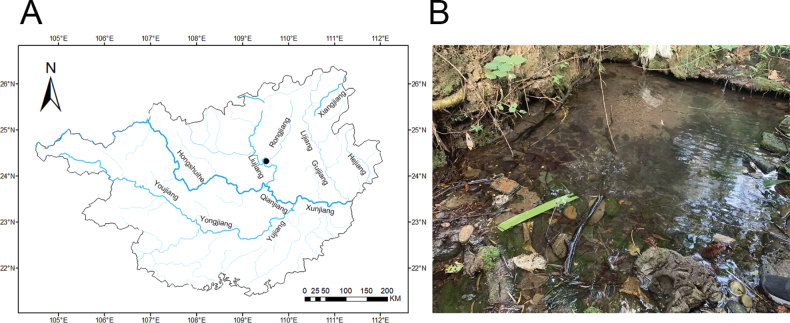
Distribution of *Oreonectes qinae* sp. nov. in Guangxi, China (A); Habitat photo of type locality at time of collection (B).

Tables 1, 2, Figs 1, 2, 3, 4

##### Type material.

***Holotype*** • KIZ2024010559, holotype, 78.6 mm SL, female; China: Guangxi Zhuang Autonomous Region: Gutingshan Forest Park, Youfeng District, Liuzhou City, Liujiang River, 24.32325°N, 109.51695°E; collected by Z.S. Qin and Y. Cai, December 2024. ***Paratypes*** • KIZ2024010560–563, four specimens, 46.3–70.5 mm SL, female; KIZ2024010564, 52.1 mm SL, male, data same as holotype.

##### Other materials.

• KIZ2024010565–571, eight specimens, 30.2–40.0 mm SL, preserved in 99% ethanol for molecular study, data same as the type specimens.

##### Etymology.

The species name, *qinae*, is in recognition of the contributions of Zi-Shan Qin to the collection of the present specimens and dedicated efforts in fieldwork. We suggest the Chinese vernacular name “覃氏岭鳅”.

##### Diagnosis.

This new species can be distinguished from all known species of *Oreonectes* by a combination of the following characteristics: color pattern present; swim bladder degenerated; black longitudinal stripe from posterior upper pectoral fin to caudal-fin base; dorsal-fin origin posterior to pelvic-fin origin; tip of pelvic fin not reaching anus; caudal fin with irregular black markings; caudal fin rounded; maxillary barbel not reaching posterior margin of opercula; without dark brown V-shaped marking on dorsal surface of head; 16 branched caudal-fin rays; and 12 inner-gill rakers on first gill arch.

##### Description.

The morphometric and meristic data of *Oreonectes qinae* sp. nov. are provided in Table 1. Body elongated and cylindrical (Fig. 1A), slight elevation of head-dorsal junction, deepest body depth 13.2%–19.8% of SL, deepest body depth anterior to dorsal-fin origin, decreasing depth from dorsal-fin origin to caudal-fin base, dorsal-fin origin closer to the posterior margin of the operculum than to caudal-fin base. Head flattened, head width longer than height, head width 60.9%–72.6% of HL, head height 43.5%–54.5% of HL. Whole body covered by scales, except head and thorax. Dorsum of head and above lateral line dark gray. Lighter color below lateral line and abdomen, without body color spots. Mouth inferior, curved, processus dentiformis absent, jaws completely covered by lips (Fig. 1H), snout length shorter than postorbital length of head, visible wrinkles present on surface of lip, lower lip with V-shaped median notch completely interrupted by longitudinal groove (Fig. 1C). Anterior and posterior nostrils closely adjacent, base of anterior nostril tubular, tip elongated to whisker-like, nostril barbel length 90.4%–140.2% of eye diameter.

**Table 1.** Morphometric and meristic data of *Oreonectes qinae* sp. nov.

Characters	Holotype (female)	Paratypes (mean ± SD)	
		Females (*N* = 11)	Males (*N* = 2)
Total length (mm)	97.9	34.9–87.2 (58.2 ± 22.5)	38.7–63.9 (51.3 ± 17.8)
Standard length (mm)	77.0	28.1–69.4 (46.3 ± 17.3)	30.9–52.4 (41.7 ± 15.2)
Percentage of standard length (%)			
Deepest body depth	17.5	11.0–16.4 (14.3 ± 1.6)	13.1–13.3 (13.2 ± 0.1)
Body width	15.5	7.8–15.8 (11.1 ± 3.0)	7.3–12.1 (9.7 ± 3.4)
Head width	17.0	14.6–16.3 (15.5 ± 0.7)	13.6–15.7 (14.7 ± 1.5)
Head depth	11.8	10.5–12.3 (11.4 ± 0.5)	10.6–10.8 (10.7 ± 0.1)
Head length	23.4	22.6–25.1 (23.9 ± 0.7)	21.8–24.3 (23.0 ± 1.7)
Predorsal length	62.7	60.0–63.9 (62.3 ± 1.1)	59.7–61.4 (60.5 ± 1.2)
Preventral length	53.4	51.5–56.4 (54.4 ± 1.5)	51.0–51.5 (51.3 ± 0.3)
Preanal length	77.9	77.6–80.4 (79.2 ± 1.0)	78.1–81.6 (79.9 ± 2.4)
Preanus length	72.9	70.6–74.1 (72.6 ± 1.2)	71.0–71.8 (71.4 ± 0.5)
Pectoral-fin length	19.5	17.1–21.0 (19.2 ± 1.1)	18.0–19.7 (18.8 ± 1.2)
Distance between pectoral fin and pelvic fin	30.7	27.1–33.8 (30.2 ± 2.1)	27.0–30.9 (28.9 ± 2.7)
Pelvic-fin length	17.0	15.5–18.0 (17.1 ± 0.8)	16.9–20.9 (18.9 ± 2.8)
Distance between pelvic fin and anal fin	24.4	21.7–26.7 (25.1 ± 1.4)	27.3–29.8 (28.6 ± 1.7)
Caudal-peduncle length	13.6	10.7–15.2 (13.1 ± 1.0)	12.2–13.3 (12.7 ± 0.7)
Caudal-peduncle depth	12.8	10.1–12.8 (11.2 ± 0.9)	10.9–11.2 (11.0 ± 0.2)
Percentage of lateral head length (%)			
Eye diameter	16.0	13.0–22.5 (18.3 ± 3.2)	17.9–18.7 (18.3 ± 0.5)
Interorbital width	45.8	36.7–46.4 (40.3 ± 3.5)	37.8–41.7 (39.8 ± 2.7)
Postorbital length	54.3	50.6–55.3 (53.3 ± 1.6)	48.7–49.9 (49.3 ± 0.8)
Snout length	30.3	30.1–36.1 (32.5 ± 2.0)	33.7–37.9 (35.8 ± 2.9)
Percentage of caudal-peduncle length (%)			
Caudal-peduncle depth	93.9	76.0–99.4 (86.5 ± 8.6)	84.5–89.1 (86.8 ± 3.2)
Counts			
Dorsal-fin rays	3, 7	3, 7–8	3, 7
Pectoral-fin rays	2, 9	2, 9–10	2, 10
Pelvic-fin rays	2, 6	2, 6–7	2, 7
Anal-fin rays	3, 5	3, 5	3, 5
Branched caudal-fin rays	16	16	16

Three pairs of barbels, well-developed, inner rostral barbel length 30.2%–39.1% of HL, reaching anterior nostril; outer rostral barbel length 47.3%–57.3% of HL, extending to posterior margin of eye; maxillary barbel length 40.8%–50.9% of HL, not reaching posterior margin of opercula. Eyes normal, interorbital width greater than eye diameter, eye diameter 13.0%–22.5% of HL. Tip of pelvic fin close to, but not reaching, anus (Fig. 1). Dorsal-fin origin posterior to pelvic-fin origin. Distance between anus and tip of anal fin 0.9–2.0 times eye diameter. Caudal fin rounded. Caudal-peduncle length greater than width, caudal-peduncle depth 76.0%–99.4% of length, adipose crests along both dorsal and ventral sides absent.

Three unbranched and seven to eight branched dorsal-fin rays, dorsal-fin rays dark, membrane transparent and scattered with black pigments, two unbranched and nine to 10 branched pectoral-fin rays, two unbranched and six to seven branched pelvic-fin rays, three unbranched and five branched anal-fin rays, 16 branched caudal-fin rays. Inner-gill rakers on first gill arch 12 (2). Cephalic lateral-line system well-developed, 9–10+4 infraorbital pores, 7–8 supraorbital canal pores, six supratemporal canal pores, 9–10 preoperculo-mandibular canal pores. Lateral line incomplete, with 11–13 lateral-line pores, last lateral-line pore not reaching tip of pectoral fin.

Posterior chamber of swim bladder degenerated, only 1.5 times eye diameter (Fig. 2A). Stomach U-shaped, intestine curved along stomach, leading directly to anus (Fig. 2B).

##### Genetic comparisons.

BI analysis based on 1116 bp of *Cytb* sequences produced consistent topologies. The phylogenetic reconstruction confirmed the validity of the new species, with high nodal support (BPP ≥ 0.95). All *Oreonectes* species formed a well-supported monophyletic group, phylogenetically resolved as the sister lineage to the clade containing *Guinemachilus* and *Micronemacheilus*. *Oreonectes qinae* sp. nov. formed a highly supported clade with *O. damingshanensis* and *O. zhangi* (Fig. 3). Uncorrected *p*-distances based on *Cytb* between *Oreonectes qinae* sp. nov. and the nine other species ranged from 1.4% (*O. damingshanensis*) to 8.7% (*O. guananensis*) (Table 2).

**Table 2.** Uncorrected *p*-distances (%) between 10 *Oreonectes* species based on cytochrome *b* (*Cytb*).

ID	Species	1	2	3	4	5	6	7	8	9
1	*Oreonectes qinae* sp. nov.									
2	*Oreonectes andongensis*	5.5								
3	*Oreonectes damingshanensis*	1.4	6.8							
4	*Oreonectes guilinensis*	6.9	6.9	7.2						
5	*Oreonectes guananensis*	8.7	7.0	8.6	8.8					
6	*Oreonectes luochengensis*	7.4	6.2	7.5	8.1	3.5				
7	*Oreonectes platycephalus*	2.2	3.9	2.0	2.0	5.6	4.9			
8	*Oreonectes polystigmus*	5.9	5.3	5.7	7.1	6.1	6.0	3.6		
9	*Oreonectes yuedongensis*	5.0	5.2	5.6	4.8	6.8	6.2	2.6	4.9	
10	*Oreonectes zhangi*	4.5	6.8	4.2	7.3	9.0	7.2	3.5	6.3	5.4

##### Sexual dimorphism.

Mature males with genital papilla positioned immediately posterior to anus; gonopore opening at tip of fleshy prominence (Fig. 1G); feature absent in females (Fig. 1C). Females generally larger than males, with pelvic-fin length measuring 17.33% of SL in female KIZ2024010559 vs. 16.9% in male KIZ2024010564.

##### Coloration.

Dorsum of head and above lateral line dark gray. Lighter color below lateral line and abdomen, without body color spots. In both sexes, distinct black stripe extending from lateral line to caudal-fin base; caudal-fin base black. In life, body dark gray except abdomen pinkish; small black spots covering body except on abdomen; no irregular spots (Fig. 1D). Dorsal fin with black pigments on fin membrane. In formalin-fixed specimens, body color, black spots, and stripe somewhat faded, dark gray above head and lateral line, light color below lateral line, abdomen white (Fig. 1A–C, E–G).

##### Distribution and habitat.

*Oreonectes qinae* sp. nov. is currently only known from Gutingshan Forest Park, Youfeng District, Liuzhou City, Guangxi Zhuang Autonomous Region, China, in the Liujiang River drainage (24.32325°N, 109.51695°E) (Fig. 4A). The species inhabits small streamlets with water depths ranging from 0.5 to 1.5 m, characterized by rocky substrate and abundant macrophyte growth, providing suitable habitat conditions (Fig. 4B). Other aquatic species observed in the same streamlet include the crab *Qianguimon splendidum* Huang, 2018, and the shrimp *Caridina huananensis* Liang, 2004; there is no other fish in the habitat.

## ﻿Discussion

The distinct phylogenetic placement of *Oreonectes qinae* sp. nov., supported by an uncorrected *p*-distance of 1.4% from *O. damingshanensis* and 4.5% from *O. zhangi*, combined with clear morphological differentiation, substantiates its recognition as a valid species.

Currently, the genus *Oreonectes* comprises 10 described species, including the newly identified species. Species of *Oreonectes* are primarily distributed in the Nanliujiang River (*O. zhangi*) and the Pearl River (*O. andongensis*, *O. damingshanensis*, *O. guananensis*, *O. guilinensis*, *O. luochengensis*, *O. platycephalus*, *O. polystigmus*, *Oreonectes qinae* sp. nov., and *O. yuedongensis*), with records spanning Guangxi, Guangdong, Hong Kong, and Quang Ninh Province in northern Vietnam (Günther 1868; Kottelat 2001; Du et al. 2008; Yang et al. 2011a; Yang et al. 2011b; Huang et al. 2020; Yu et al. 2023; Luo et al. 2024; Zhong et al. 2024; Lan et al. 2024). Within the genus, *O. damingshanensis*, *O. platycephalus*, *O. qinae* sp. nov., and *O. zhangi* are characterized by a degenerated swim bladder, while *O. andongensis*, *O. guananensisO. guilinensis*, and *O. luochengensis* are characterized by a well-developed swim bladder. Therefore, *O. qinae* sp. nov. can be distinguished by its swim bladder morphology from other congeners, except for *O. damingshanensis*, *O. platycephalus*, and *O. zhangi*.

However, *O. qinae* sp. nov. can be readily distinguished from *O. damingshanensis* (sister species on phylogenetic trees, *p*-distances 1.4% with new species) by 16 branched caudal-fin rays (vs. 14), 11–13 lateral-line pores, last pore not reaching tip of pectoral fin (vs. 14–15 lateral-line pores, last reaching above tip of pectoral fin), 12 inner-gill rakers on first gill arch (vs. nine), without irregular black spots present on dorsal and lateral surfaces (vs. irregular black spots present on dorsal and lateral surfaces), a short tube connected two chamber of air-bladder (vs. a long and slender tube), and the new species is primarily found in Liuzhou City, Guangxi, belonging to the Liujiang River basin of the Pearl River system (Nanning City, Guangxi, belonging to the Hongshuihe River basin of the Pearl River system), Although the two species share only 1.4% genetic divergence, their pronounced morphological disparity and distinct geographic ranges provide unequivocal evidence for their recognition as separate species. A comparable pattern is observed in *Traccatichthys pulcher* Nichols & Pope, 1927 and *Traccatichthys taeniatus* Pellegrin & Chevey, 1936, which exhibit a low genetic distance of 1.58% (Qin et al. 2025).

Compared with congeners characterized by a degenerated swim bladder, *Oreonectes qinae* sp. nov. can be distinguished from *O. platycephalus* by 16 branched caudal-fin rays (vs. 14–15), black longitudinal stripe from posterior upper pectoral fin to caudal-fin base (vs. short black stripe), and head width 60.9%–72.6% of HL (vs. 79.0%–84.6%); from *O. zhangi* by the tip of the pelvic fin not reaching anus (vs. surpassing), a black longitudinal stripe from posterior upper pectoral fin to caudal-fin base (vs. short black stripe), 12 inner-gill rakers on first gill arch (vs. nine), head length 21.8%–25.1% of SL (vs. 18.6%–20.2%), predorsal length 59.7%–63.9% of SL (vs. 56.2%–59.4%), prepelvic length 51.0%–56.4% of SL (vs. 48.1%–50.8%), preanal length 77.6%–81.6% of SL (vs. 67.7%–72.1%), snout length 30.1%–37.9% of HL (vs. 38.2%–48.8%), and postorbital length 48.7%–55.3% of HL (vs. 43.0%–47.9%).

All morphological and molecular evidence supported the validity of the new species. The presence of oocytes in the abdominal cavities of female specimens indicated that individuals of *Oreonectes qinae* sp. nov. were undergoing active reproductive development at the time of collection. Among the known species of *Oreonectes*, except the four species collected in caves, those collected from surface streams, including *O. damingshanensis* and *O. zhangi* in February, *O. platycephalus* in April, *O. guilinensis* in May, *O. yuedongensis* in July, and *Oreonectes qinae* sp. nov. in December, were either in the reproductive phase or approaching it. The nutritional constraints of karst cave ecosystems, characterized by limited food availability, impose significant challenges for sustaining the metabolic demands associated with reproduction (Chen et al. 1994). Based on the present findings, we propose that species of *Oreonectes* predominantly inhabit subterranean environments or in comparable dark microhabitats (e.g., beneath thick layers of decaying leaf litter) throughout most of their life cycles, emerging into surface habitats primarily during the reproductive season. The habitat occupied by *Oreonectes qinae* sp. nov. appears to have been formed during flood events, suggesting that this species likely migrates from underground rivers to surface streams coinciding with flood periods to facilitate reproduction.

The tectonic uplift of the Tibetan Plateau played a pivotal role in shaping the Pearl River system (Zhang et al. 2022), establishing extensive surface water networks that facilitated dispersal and diversification within the genus *Oreonectes* (Luo et al. 2024). Nowadays, *Oreonectes* exhibits a broad distribution across Guangdong, Guangxi and northern Vietnam. The combination of complex karst landforms in Guangxi, the small body size of these fish, and their limited mobility has driven repeated episodes of speciation and subspeciation (Lan et al. 2024). Minimal morphological differentiation among species, together with the prevalence of cryptic diversity, continues to complicate taxonomic classification within *Oreonectes*.

### ﻿Comparative material

All specimens for comparison are as follows:

*Oreonectes andongensis*, GXNU20220601, holotype, 60.1 mm SL, GXNU20220602–10, paratypes, 9 ex., 36.8–56.2 mm SL, Andong Town, Xincheng County, Laibin City, Hongshui River system, Guangxi.

*Oreonectes damingshanensis*, GZNU20230216001, holotype, 81.8 mm SL, GZNU20230216002–010, 52.5–70.7 mm SL, GZNU20230216012–013, 60.8–61.3 mm SL, GZNU20230216015, 52.8 mm SL, GZNU20230216016–20, 54.3–64.1 mm SL, GZNU20230216022–25, 46.2–48.9 mm SL, paratypes, 21 ex., Mashan Town, Nanning City, Guangxi.

*Oreonectes guananensis*, KIZ2010003067, holotype, 72.9 mm SL, KIZ2010003068–072, paratypes, 5 ex., 50.6–71.9 mm SL, Guan’an Village, Changmei Town, Huanjiang County, Guangxi.

*Oreonectes guilinensis*, ASIZB208001, holotype, 73.9 mm SL, ASIZB208002–007, paratypes, 6 ex., 66.6–82.9 mm, Shigumen Village Xingping Town, Yangshuo County, Guilin City, Guangxi.

*Oreonectes luochengensis*, KIZ2010003073, holotype, 71.5 mm SL, KIZ2010003074–077, KIZ2010003242–244, paratypes, 7 ex., 61.5–76.4 mm SL, Tianhe Town, Luocheng County, Guangxi.

*Oreonectes platycephalus*, GZNU20230701013, 53.5 mm SL, GZNU20230701015–21, 27.3–54.9 mm SL, Guangzhou City, Guangxi. GZNU20230701022–23, 44.1–50.9 mm SL, GZNU20230701025, 43.2 mm SL, 11 ex., Hong Kong University.

*Oreonectes polystigmus*, KIZ2001004626, holotype, 56.4 mm SL, KIZ2002004627–634, paratypes, 8 ex., 34.5–53.2 mm SL, Dabu Village, Guilin City, Guangxi.

*Oreonectes yuedongensis*, GZNU20230304001, 60.2 mm SL, GZNU20230304003–006, 54.3–42.5 mm SL, GZNU20230304008, 43.1 mm SL, GZNU20230325003, 42.8 mm SL, GZNU20230409001–002, 37.5–39.7 mm SL, paratypes, 9 ex., Puning Town, Jieyang City, Guangdong.

*Oreonectes zhangi*, NNNU2023100203, holotype, 60.9 mm SL, NNNU2023100207, 61.6 mm SL, NNNU2023100210, 67.3 mm SL, NNNU2023100212–13, 48.4–55.2 mm SL, paratypes, 4 ex., Xinye County, Yulin City, Guangxi.

## Supplementary Material

XML Treatment for Oreonectes qinae

## ﻿Additional information

### Use of AI

No use of AI was reported.

### Author ORCIDs

Zhuo-Ni Chen https://orcid.org/0009-0003-7209-4421

Li-Na Du https://orcid.org/0000-0002-2246-643X

### Data availability

All of the data that support the findings of this study are available in the main text.
